# Landmark Regression with Attention U-Net for Assessing Breast Positioning Quality in MLO Mammography View

**DOI:** 10.3390/diagnostics16142262

**Published:** 2026-07-20

**Authors:** Nurper Denizoglu, Toygar Tanyel, Enes Can Erkan, Mustafa Ege Seker, Emine Meltem, Esma Aktufan Cerekci, Deniz Alis, Ilkay Oksuz, Ercan Karaarslan, Erkin Aribal

**Affiliations:** 1 Department of Radiology, Sultan 2. Abdulhamid Han Training and Research Hospital, Istanbul 34668, Turkey; 2 Biomedical Engineering Graduate Program, Istanbul Technical University, Istanbul 34469, Turkey; 3 Department of Radiology, School of Medicine, University of Wisconsin, Madison, WI 53792, USA; 4 Department of Radiology, Istanbul Training and Research Hospital, University of Health Sciences, Istanbul 34098, Turkey; 5 Department of Radiology, Sisli Hamidiye Etfal Training and Research Hospital, Istanbul 34371, Turkey; 6 Department of Radiology, School of Medicine, Acibadem Mehmet Ali Aydinlar University, Istanbul 34684, Turkey; 7 Department of Computer Engineering, Istanbul Technical University, Istanbul 34469, Turkey

**Keywords:** breast cancer, mammography, deep learning, positioning quality

## Abstract

**Background/Objectives:** This study developed and evaluated a novel Attention U-Net regression model to assess MLO mammography positioning quality, comparing it against a plain U-Net regression baseline (architectural ablation) and a ResNeXt50 classification baseline. **Methods:** We curated 1000 patient mammograms (2000 MLO images) from the public VinDr-Mammo dataset, with pectoral muscle line and nipple positions annotated by an expert breast radiologist. Three deep learning models were compared under a 10-fold stratified cross-validation protocol. Statistical significance was assessed with paired Wilcoxon signed-rank tests, McNemar’s test, Cochran’s Q, Wilson score confidence intervals, bootstrap confidence intervals, Holm–Bonferroni correction, and paired Cohen’s d effect sizes. **Results:** Against the automated PNL reference, the Attention U-Net achieved 85.5% accuracy (Wilson 95% CI [83.9%, 87.0%]), 80.2% sensitivity, and 88.7% specificity, outperforming both the plain U-Net (71.8% accuracy) and ResNeXt50 (73.7% accuracy). (Cochran’s Q *p* < 0.001; pairwise McNemar *p* < 0.001). For landmark localization, the Attention U-Net produced significantly lower errors across all five evaluated landmark and geometric measures (nipple 3.44 mm vs. 7.02 mm; perpendicular intersection 5.93 mm vs. 9.81 mm; all comparisons remained significant after Holm–Bonferroni correction). **Conclusions:** In this single-dataset, single-view study, an Attention U-Net landmark-regression model provided explicit landmark and PNL outputs for quantitative MLO mammography positioning assessment. The model showed improved accuracy and sensitivity compared with a plain U-Net regression baseline and a ResNeXt50 classification baseline.

## 1. Introduction

Breast cancer is the most prevalent form of cancer and the leading cause of cancer-related deaths among women worldwide, according to the World Health Organization [1]. Mammography screening has been established as the most effective method for the early diagnosis of breast cancer, significantly reducing mortality rates [2]. Consequently, many countries have implemented national breast cancer screening programs [3].

A standard screening mammogram typically includes the craniocaudal (CC) and mediolateral oblique (MLO) views of each breast. The MLO view is particularly critical as it can display nearly the entire breast tissue when performed correctly. Moreover, this view prominently features the upper quadrant of the breast, which is the most common site for breast cancer occurrences.

Mammography positioning is crucial for accurate readings, as poor positioning is a common cause of diagnostic deficiencies and misdiagnoses in mammography screenings [4,5,6,7]. Incorrectly positioned mammograms may necessitate re-examinations, thereby incurring additional costs and causing emotional distress for women recalled for repeat examinations. Consequently, there is an unmet need for automated systems capable of assessing positioning quality instantly. Such systems would alert radiology technologists to consult an expert radiologist or repeat the acquisition immediately after the initial mammography.

In recent years, the application of deep learning (DL) to breast cancer diagnostics has grown extensively, with several studies demonstrating that DL can perform on par with, or even surpass, radiologists in detecting breast cancer [8,9]. Despite numerous studies applying DL to breast cancer diagnosis in mammography, relatively less effort has been invested in using DL to automatically assess mammography positioning. Such applications have the potential to automate and streamline the assessment of mammography positioning immediately after acquisition. Most earlier studies have relied on classification-based DL approaches, using qualitative assessments by experts as the reference. This approach potentially hinders the explainability and objectivity of the results provided by the DL systems [10,11].

U-Net is a widely used convolutional neural network designed for biomedical image segmentation, featuring an encoder–decoder architecture with skip connections to preserve spatial information [12]. Attention U-Net extends this by incorporating attention gates, which help the model focus on the most relevant anatomical structures—such as the pectoralis muscle and nipple—while filtering out irrelevant background noise [13]. This mechanism improves localization accuracy by focusing on the most relevant anatomical features, supporting a more reliable assessment of positioning quality; it is a feature-refinement mechanism and does not, by itself, make the network’s internal decision process explainable.

In this work, we propose a novel regression-based deep learning approach validated on a single public dataset using an Attention U-Net to quantitatively evaluate MLO mammography positioning. The main contributions of this study are as follows:

• We developed a landmark-regression model using an Attention U-Net architecture to localize the nipple and pectoral muscle endpoints, enabling automated calculation of the PNL.
• We designed the model output to provide explicit landmarks, a derived PNL, and a visible PNL intersection point, supporting quantitative and output-level interpretable assessment of MLO positioning quality.
• We added a matched plain U-Net regression baseline as an architectural ablation to evaluate the contribution of attention gates to localization and downstream positioning-quality classification.
• We compared the proposed regression-based approach with a ResNeXt50 classification baseline under automated PNL and expert qualitative references, reporting confidence intervals, paired statistical tests, and effect sizes.

## 2. Materials and Methods

### 2.1. Study Sample

This study used the publicly available VinDr Mammography open-access large-scale dataset [13]. The VinDr Mammography dataset includes 5000 mammography exams collected from opportunistic screening settings of two hospitals in Vietnam and mammography devices from three different vendors (Siemens—Erlangen, Germany, IMS—Madrid, Spain, Planmed—Helsinki, Finland) between 2018 and 2020. Since this dataset is publicly available and all patient data was fully anonymized prior to release, no additional institutional review board approval was required for this study. The original dataset creators ensured compliance with ethical and legal guidelines for data sharing and research use. Accordingly, no new patient recruitment, data collection, or direct patient interaction occurred in this study. This exemption aligns with international ethical standards for secondary analysis of de-identified public datasets.

DL models perform best with abundant data, especially when training data is comprehensive [14]. However, annotating medical images is time-consuming and computationally intensive. To optimize our limited resources, we randomly selected 1000 out of 5000 exams. Each exam includes two MLO-view mammograms from both breasts. Two thousand mammography images from 1000 patient examinations were evaluated using a randomized 10-fold patient-level grouped stratified cross-validation. Both MLO views from the same patient/examination were assigned to the same fold to prevent train–test overlap within a patient. Cross-validation folds were stratified by the good/poor positioning label, while preserving the per-image good/poor distribution across folds as closely as possible.

### 2.2. Image Positioning Quality Criterion

Numerous international mammography quality evaluation systems have been proposed, each offering a distinct yet overlapping set of positioning criteria for MLO views. These criteria include the angle of the pectoral muscle, its width and length, the angulation of the anterior and posterior borders of the pectoral muscle, the distance of the inferior aspect of the pectoral muscle relative to the nipple level, and the posterior nipple line (PNL) drawn from the nipple to the pectoralis muscle.

Despite the variety in quality criteria, the primary goal remains to capture as much breast tissue as possible in the MLO view. However, some criteria, such as the distance from the inferior aspect of the pectoral muscle to the nipple level, are highly subjective and often unachievable [15]. Others, like the pectoral muscle angle and the dimensions of the pectoral muscle, lack established standards for what constitutes optimal positioning.

A consistent, achievable, and reliable criterion to ensure the greatest amount of breast tissue coverage is that the PNL, drawn from the nipple to the pectoralis muscle at a perpendicular angle, should touch the pectoralis muscle rather than the edge of the film. This approach is endorsed by both the American College of Radiology and the Royal Australian and New Zealand College of Radiologists [15,16,17,18,19]. Accordingly, we have selected this criterion as the reference standard for the current study.

### 2.3. Ground Truthing Process

Accurate reference data was necessary to train and evaluate our model, often called ‘ground truth’. Ground truth annotations were carried out by a board-certified breast radiologist (N.D.), with over five years of experience in breast imaging. The radiologist marked mammograms using a specialized workstation that included a browser-based annotation tool (https://md.ai, accessed on 1 June 2026) and a 6-megapixel diagnostic monitor (Radiforce RX 660, EIZO, Hakusan, Japan). All mammograms were reviewed in the Digital Imaging and Communications in Medicine format.

**Figure 1:**
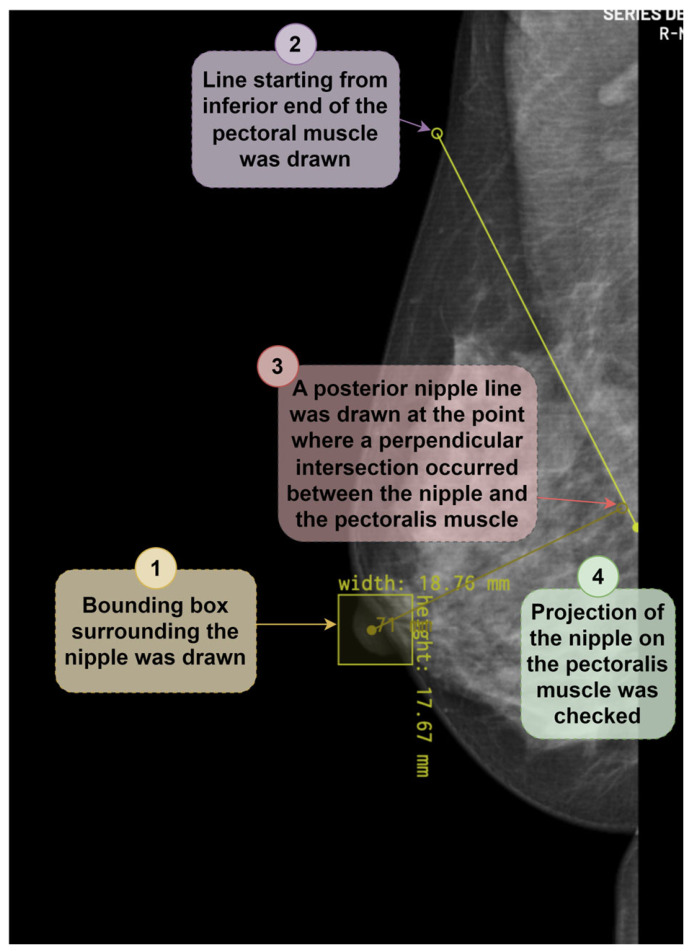
Illustration of the ground-truth annotation protocol for MLO views. (1) A bounding box is annotated around the nipple. (2) The pectoral muscle line is defined by marking its superior and inferior extents. (3) The posterior nipple line (PNL) is derived by extending a perpendicular line from the nipple to the pectoral muscle. (4) Quality is determined by the intersection point: if the PNL intersects the muscle, the image is ‘Good’; if it intersects the posterior image boundary (missing the muscle), it is ‘Poor’.

The radiologist labeled the nipple, pectoralis muscle line and perpendicular line from nipple to pectoral muscle on MLO views. Figure 1 exemplifies the criterion used for assessing image positioning in MLO views.

### 2.4. Image Preprocessing

DICOM files from VinDr-Mammo were parsed with pydicom; pixel arrays were extracted along with PixelSpacing metadata to enable evaluation in physical (millimeter) units. Landmark annotations exported from MD.ai (nipple bounding box centroid, pectoral muscle line endpoints) were converted into pixel coordinates aligned with the DICOM image grid. Preprocessing then proceeded as follows: (i) breast tissue isolation via thresholding and largest-connected-component selection to crop peripheral background; (ii) zero-padding to preserve the original aspect ratio; (iii) resizing to 512 × 512 pixels using bilinear interpolation, with landmark coordinates rescaled by the same factor; (iv) intensity normalization to the [0, 1] range; (v) endpoint adjustment of the pectoral muscle line to align with image boundaries with a 10-pixel safety margin to prevent edge-related errors. Pixel spacing was preserved through all transformations to allow conversion of pixel-space distances to millimeters at evaluation time. Predicted landmark coordinates produced by the model in the resized 512 × 512 pixel space were subsequently converted to physical millimeter units using the original DICOM PixelSpacing metadata combined with the resize ratio. The complete pipeline is available in the GitHub repository (https://github.com/enescanerkan/deep-breast-positioning, accessed on 1 June 2026).

### 2.5. Attention Regression U-Net Model

To evaluate breast positioning, we developed a model employing an attention-enhanced U-Net architecture, a common advanced AI model used for medical imaging tasks. This model is structured into two main components, encoder and decoder, with attention gates.

The encoder summarizes key, high-level features of the mammogram (such as morphological patterns, boundaries, and textural information of the breast anatomy) by progressively compressing the input image through a series of convolutional layers and pooling operations. These operations reduce spatial dimensions while preserving essential feature representations.

The decoder expands the compressed feature maps to reconstruct the image detail, focusing on important areas (e.g., the nipple) with the help of attention gates. These gates are integrated at each stage of the decoder to help the model “pay attention” to relevant features while suppressing less critical information. The model provides output-level interpretability through explicit predicted landmarks, the derived PNL, and the visible PNL intersection point. This should be distinguished from mechanistic explainability of the network’s internal representations. Attention gates are used as a feature-refinement mechanism to support localization, but the clinically interpretable component is the explicit geometric output.

The model outputs the coordinates of the landmarks through a regression approach, where a final convolution layer predicts the necessary coordinates. This structure allows for a detailed yet efficient analysis of mammographic positioning quality. The attention mechanisms specifically help in maintaining focus on relevant features while ignoring distracting or irrelevant regions, which is crucial for maintaining high accuracy in landmark detection for medical imaging.

Architecturally, the Attention U-Net follows a 5-level encoder–decoder structure with skip connections gated by attention modules. The encoder uses base channel widths of 64, 128, 256, 512, and 1024 across the five levels; each level comprises two 3 × 3 convolutional blocks (Conv → BatchNorm → ReLU). Downsampling between levels is performed by 2 × 2 max-pooling. The decoder mirrors the encoder with transposed-convolution upsampling (2 × 2, stride 2) and concatenates the corresponding gated skip-connection feature map at each level. Each attention gate is a soft additive-attention module that computes a sigmoid-activated coefficient map from the gating signal (decoder feature map) and the skip signal (encoder feature map), with intermediate channel dimension equal to half the skip-signal channel count. The final output is a 1 × 1 convolution producing six channels corresponding to the (x, y) coordinates of the three anatomical landmarks. The plain U-Net baseline used for architectural ablation has the identical encoder–decoder topology with all attention gates removed.

**Figure 2:**
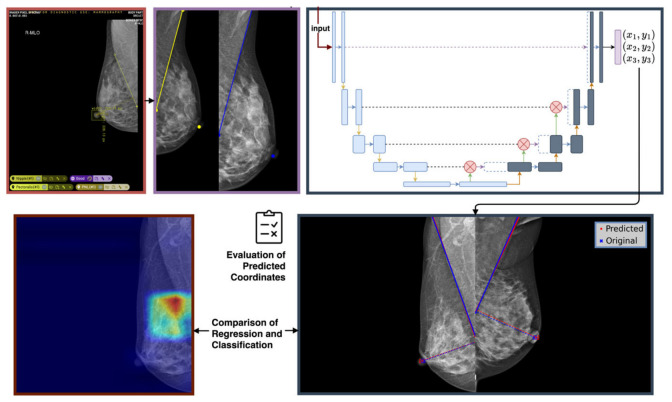
Schematic overview of the deep learning pipeline. This figure illustrates the study workflow, beginning with expert radiologist annotations of the nipple and pectoral muscle using a dedicated browser-based annotation platform (top-left panel). Next, image preprocessing is performed to remove non-breast regions, enhancing feature extraction (top-middle panel). The dataset is then split into training, validation, and testing sets for model development using U-Net-based approaches (top-right panel). The Attention U-Net model (bottom-left panel) predicts anatomical landmarks to assess positioning, while the ResNeXt50 model (bottom-right panel) classifies images as well or poorly positioned. The outputs from both models are evaluated and compared using the posterior nipple line (PNL) criterion to determine image quality in MLO views.

In training our Attention U-Net model, specialized hardware, a single NVIDIA RTX A6000 GPU, was used. We configured it with a single input channel and six output channels to map the x and y coordinates of three anatomical landmarks. To optimize the model’s performance training was performed over 300 epochs. The model used a mathematical technique called Wing Loss, to learn how to identify landmarks, which helps it account for small variations in complex images. The most effective model, determined by the lowest validation loss, was selected for further evaluation and potential clinical application. The DL pipeline used in this study is shown in Figure 2.

### 2.6. Training Configuration

All three models were trained on a single NVIDIA RTX A6000 GPU. The Attention U-Net and plain U-Net regression models were trained with the Adam optimizer (batch size 8, 300 epochs) using a Cyclic Learning Rate schedule (base = 1 × 10^−5^, max = 5 × 10^−4^). A per-landmark weighted Wing Loss was employed to balance robustness against landmark outliers with sub-pixel localization precision. The ResNeXt50 baseline classification model was trained with the Adam optimizer (batch size 8, 30 epochs) using a Cyclic Learning Rate schedule (base = 1 × 10^−5^, max = 5 × 10^−4^) with Cross-Entropy Loss. Adam was used for the regression models for stable convergence of dense coordinate regression under the Wing-based loss, while a shorter training schedule sufficed for the classification baseline given the use of ImageNet pre-training. The full training configuration is summarized in Table 1.

### 2.7. Baseline ResNeXt50 Classification Model

In addition to identifying landmarks, a second AI model was developed to categorize mammograms as either well or poorly positioned. To achieve this, we utilized a ResNeXt50 model, a streamlined system that leverages pre-existing knowledge from ImageNet, a large image database. Training was conducted on an NVIDIA RTX A6000 GPU with a batch size of 8 over 30 epochs to fine-tune the model. During training, the model learned to recognize patterns in positioning quality by analyzing previously labeled image examples. The model’s accuracy was evaluated using metrics such as loss (Categorical Cross-Entropy Loss) and performance scores (F1 score, sensitivity, and specificity), ensuring the best-performing version was saved for further use. We qualitatively evaluated how the model arrives at its decision by using widely utilized saliency-based explainable AI (XAI) method—Gradient-weighted Class Activation Mapping (Grad-CAM) [20].

### 2.8. Model Evaluation

We defined Quantitative Assessment as a binary classification derived strictly from the model’s geometric output: if the calculated PNL intersected the pectoral muscle line coordinates, the image was classified as ‘Good’; if the intersection point fell on the posterior image boundary (missing the muscle), it was classified as ‘Poor’. Specifically, we wrote a function that calculates the intersection point of a perpendicular line drawn from the nipple coordinates $\left(x_{n},y_{n}\right)$ to the pectoral muscle line, which is defined by the endpoints $\left(x_{1},y_{1}\right)$ and $\left(x_{2},y_{2}\right)$. The function $P\left(x_{1},y_{1},x_{2},y_{2},x_{n},y_{n}\right)$, efficiently processes lines with arbitrary slopes by calculating the slope and intercept of the perpendicular line and analytically determining the coordinates of the intersection.

Conversely, Qualitative Assessment was defined as a holistic clinical grading performed by an expert radiologist (E. A. C.) with over ten years of experience interpreting hundreds of mammography examinations annually, adhering to American College of Radiology (ACR) quality standards [19], accounting for nuances such as muscle convexity and adequate tissue inclusion that simple geometric lines may miss. While our quantitative approach is robust, we acknowledge potential discrepancies with clinical assessments. A few millimeters’ deviation from the pectoral muscle, though classified as ‘poor’ by our model, may still be deemed diagnostic by experienced radiologists.

### 2.9. Statistical Analyses

All statistical analyses were implemented in Python (v3.9) using SciPy (v1.13.1), NumPy (v1.26.4), and statsmodels. AI models were evaluated under a 10-fold patient-level grouped stratified cross-validation protocol, ensuring that both MLO views from the same patient/examination were kept in the same fold while preserving balanced ‘good’ and ‘poor’ label distributions in each test partition. Standard performance metrics were derived from the confusion matrix as Accuracy = (TP + TN)/(TP + TN + FP + FN), Sensitivity = TP/(TP + FN), and Specificity = TN/(TN + FP). Sensitivity is computed for the ‘poor’ (positive) class consistent with the clinical detection task, and specificity for the ‘good’ class.

Cross-validation splits were generated at the patient/examination level rather than the image level. The patient/examination identifier was used as the grouping variable so that the left and right MLO views from the same examination could not appear in different folds. The stratification variable was the qualitative Good/Poor positioning label, and folds were generated to preserve the overall per-image Good/Poor distribution across the 2000-image pool as closely as possible. For classification metrics we report Wilson score 95% confidence intervals at the per-image level (*n* = 2000 MLO-view images pooled across patient-level grouped folds), together with paired Wilcoxon signed-rank tests and paired Cohen’s d effect sizes for fold-level model comparisons.

For landmark localization, predictions in the resized 512 × 512 pixel space were converted to physical millimeter units using the original DICOM PixelSpacing metadata. Per-landmark mean Euclidean errors are reported with bootstrap 95% confidence intervals (10,000 resamples), paired Wilcoxon signed-rank tests, and paired Cohen’s d for the U-Net vs. Attention U-Net comparison. Because multiple pairwise comparisons were performed, *p*-values from related hypothesis-test families were adjusted using the Holm–Bonferroni method. Both unadjusted and Holm-adjusted *p*-values are reported where applicable, and statistical significance was interpreted using the adjusted *p*-values.

## 3. Results

We employed two complementary approaches to evaluate MLO-view positioning quality. In the quantitative assessment, scans were classified based on the mathematically derived PNL intersection point. In contrast, the qualitative assessment relied on the expert radiologist’s comprehensive review of the image, integrating the PNL check with overall positioning quality cues. This dual strategy allowed us to compare an objective, measurement-driven assessment with a more holistic, radiologist-based judgment.

Based on the PNL, the cross-validation pool of 2000 MLO images was identified as 1253 ‘good’ and 747 ‘poor’. The expert’s qualitative assessment of the same images yielded 1463 ‘good’ and 537 ‘poor’.

### 3.1. Landmark Localization Performance

Pooled across the ten cross-validation folds (*n* = 2000 test images), the Attention U-Net achieved substantially lower errors than the plain U-Net across all five evaluated landmark and geometric measures (three anatomical landmarks, the derived perpendicular intersection, and pectoral-line angular agreement). The largest improvements were observed at the nipple (3.44 mm vs. 7.02 mm; mean difference 3.58 mm, 95% CI [3.38, 3.78]; paired Wilcoxon *p* < 0.001; paired Cohen’s d = 0.80), the perpendicular intersection (5.93 mm vs. 9.81 mm; mean difference 3.88 mm, 95% CI [3.64, 4.13]; *p* < 0.001; d = 0.68), and the inferior pectoral endpoint (8.10 mm vs. 12.86 mm; mean difference 4.76 mm, 95% CI [4.37, 5.15]; *p* < 0.001; d = 0.54). All five comparisons reached statistical significance after Holm–Bonferroni correction (Table 2). These localization differences are interpreted primarily through their effect on downstream positioning classification rather than as clinically decisive in isolation.

### 3.2. Classification Performance Against the Automated PNL Reference

Under patient-level grouped cross-validation, the Attention U-Net achieved 85.5% accuracy (Wilson 95% CI [83.9%, 87.0%]) against the automated PNL reference, compared with 71.8% [69.7%, 73.7%] for the plain U-Net and 73.7% [71.7%, 75.6%] for the ResNeXt50 baseline (Table 3). After Holm–Bonferroni correction of fold-level paired comparisons, the Attention U-Net remained significantly superior to both alternatives for overall accuracy and sensitivity (vs. U-Net: accuracy adjusted *p* = 0.008, sensitivity adjusted *p* = 0.016; vs. ResNeXt50: accuracy adjusted *p* = 0.008, sensitivity adjusted *p* = 0.016). ResNeXt50 attained high specificity (88.9%) but low sensitivity (48.2%), reflecting a bias toward classifying images as well-positioned; the Attention U-Net retained comparable specificity (88.7%) while substantially improving sensitivity (80.2%).

**Figure 3:**
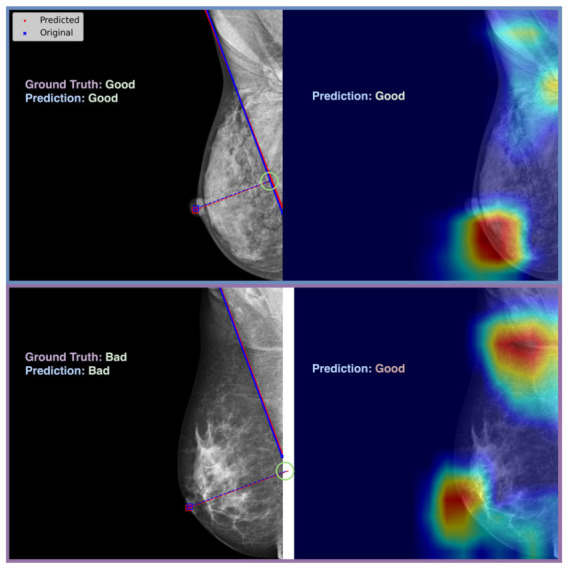
Visual comparison of model predictions on representative test cases. Top Row: A well-positioned MLO view. The Attention U-Net (**left**) accurately localizes the PNL (red line) crossing the pectoral muscle, while the ResNeXt50 (Right) correctly classifies it as ‘Good’ with relevant Grad-CAM activation. Bottom Row: A poorly positioned MLO view. The Attention U-Net (**left**) correctly plots the PNL missing the muscle (indicating ‘Poor’ quality), whereas the ResNeXt50 (**right**) incorrectly classifies the image as ‘Good’, with Grad-CAM highlighting irrelevant tissue in this representative example.

**Figure 4:**
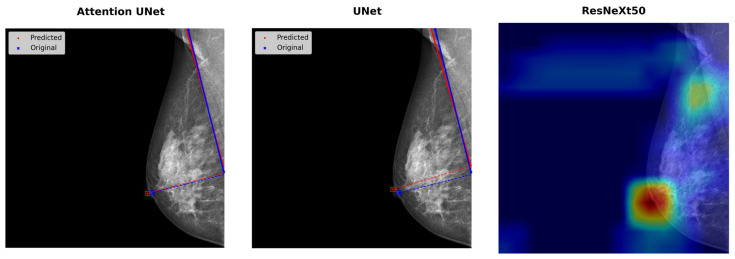
Architectural ablation: visual comparison of model outputs on a representative test case. The Attention U-Net (**left**) accurately localizes both the pectoral muscle line and the nipple, with the predicted PNL closely matching the ground truth. The plain U-Net (**middle**) without attention gates shows visible coordinate drift, illustrating the contribution of the attention mechanism to localization precision. The ResNeXt50 classification baseline (**right**) provides only a Grad-CAM activation map without explicit landmark output, with attention focused on irrelevant tissue regions in this representative example.

Representative examples illustrating the performance of both the proposed and baseline models in determining image positioning on an MLO-view mammography examination are provided in Figure 3 and Figure 4. We also qualitatively evaluated the explainability of the outputs from the baseline classification model by generating Grad-CAM activation maps for a sample of mammograms. A radiologist then visually inspected these maps to determine whether the highlighted regions aligned with clinically relevant anatomical landmarks and established positioning criteria. When the regions of interest in the Grad-CAM maps overlapped with expected anatomical features, the model’s output was considered more interpretable. However, in several instances, the model focused on irrelevant areas, indicating shortfalls in explainability. Examples of these analyses, including Grad-CAM visualizations, are provided in Supplementary Materials.

### 3.3. Classification Performance Against the Expert Qualitative Reference

When evaluated against the expert radiologist’s holistic assessment under patient-level grouped cross-validation, the Attention U-Net achieved 82.2% accuracy with 85.8% sensitivity, again outperforming both the plain U-Net (72.4%) and ResNeXt50 (78.0%) in overall accuracy (Table 4). Fold-level paired comparisons after Holm–Bonferroni correction showed that the Attention U-Net retained significant accuracy and sensitivity advantages over both alternatives (vs. U-Net: accuracy adjusted *p* = 0.0078, sensitivity adjusted *p* = 0.0156; vs. ResNeXt50: accuracy adjusted *p* = 0.0098, sensitivity adjusted *p* = 0.0156). As expected, ResNeXt50 showed higher specificity (86.3%) than the Attention U-Net (80.9%) but lower sensitivity for poorly positioned images (55.5% vs. 85.8%).

### 3.4. Statistical Comparison

At the fold level, paired comparisons across the ten cross-validation folds confirmed the per-image findings after correction for multiple testing. Nominal paired Wilcoxon *p*-values, Holm-adjusted *p*-values, and paired Cohen’s d effect sizes are summarized in Table 5. Under the automated PNL reference, the Attention U-Net retained significant accuracy and sensitivity advantages over both baselines after Holm–Bonferroni correction. Under the expert qualitative reference, the Attention U-Net also retained significant accuracy and sensitivity advantages over both baselines after correction. The correction did not alter the main conclusions. The Attention U-Net’s accuracy advantage over both alternatives was statistically significant (*p* = 0.002 for both pairwise comparisons under the automated reference; *p* = 0.004 vs. U-Net and *p* = 0.010 vs. ResNeXt50 under the manual reference) with very large effect sizes for accuracy and sensitivity (Cohen’s d > 1.1). Specificity differences were comparatively small or absent—most notably, Attention U-Net and ResNeXt50 attained nearly identical specificity under the automated reference (88.7% vs. 88.9%; d = −0.07). The plain U-Net and ResNeXt50 did not differ significantly on accuracy (Holm-adjusted *p* = 0.211 automated; *p* = 0.146 manual). Across the ten cross-validation folds, the paired Wilcoxon signed-rank test attains a minimum two-sided *p*-value of 2/2^10^ ≈ 0.00195 when all fold-level differences share the same sign; reaching this floor indicates uniformly directional fold-level superiority rather than a precise probability estimate.

## 4. Discussion

We designed a system that couples a neural landmark predictor, an attention regression U-Net, with a transparent, rule-based geometric decision: the predicted nipple and pectoral-muscle landmarks are used to derive the posterior nipple line, whose intersection with the pectoral muscle line determines positioning quality. The system therefore provides output-level, geometric interpretability, namely explicit and clinically verifiable landmark and PNL outputs, together with a transparent decision rule; it does not, however, claim mechanistic explainability of the network’s internal computation, which remains opaque as in other deep neural networks. Our findings indicate that the proposed model demonstrated consistently higher performance metrics compared to the baseline classification model when evaluated using both the PNL criterion and qualitative assessment by an expert radiologist.

Unlike classification-based models, which emit an opaque score and require post hoc attribution, our landmark-regression approach yields explicit, directly verifiable geometric outputs, allowing a clinician to inspect the predicted landmarks and the derived PNL and to judge their correctness against the anatomy. We emphasize that this is interpretability at the level of the model’s outputs rather than an explanation of its internal decision process. The ResNeXt50 classification model, while performing reasonably well, relied on a post hoc saliency technique (Grad-CAM) to interpret its results. However, saliency-based methods such as Grad-CAM have known limitations in reliably attributing decision-making to clinically relevant features, as documented in prior studies [21].

The contribution of the attention mechanism can be isolated by comparing the plain U-Net regression model to the ResNeXt50 classification baseline. These two models did not differ significantly in accuracy under the automated PNL reference (71.8% vs. 73.7%; McNemar *p* = 0.21; paired Cohen’s d = −0.25 across folds). Only with the addition of attention gates does the regression framework substantially outperform the classification baseline, suggesting that the attention mechanism is the key architectural component driving the observed performance gains rather than the regression formulation alone (Figure 4). We acknowledge that further exploration of alternative loss functions and regression backbones would enrich this analysis, and we leave such extensions to future work.

Our evaluation further compared the model’s outputs with expert radiologist assessments, acknowledging potential discrepancies due to clinical flexibility in positioning assessments. Specifically, small deviations (e.g., a few millimeters from the pectoral muscle), which our model flagged as ‘poor positioning’, may still be considered diagnostically acceptable by experienced radiologists. However, this sensitivity is advantageous when the system is deployed as a decision-support tool rather than a rigid gatekeeper. By visually projecting the PNL and its intersection point on the image, the model provides the radiographer with objective, quantitative data. This allows the clinical staff to apply their expertise to determine if a specific deviation—though mathematically ‘poor’—is clinically negligible, thereby preventing unnecessary recalls while maintaining high standards. Moreover, difference in criteria explains the noticeable shift in label distribution, where the expert identified approximately 10 percentage points more ‘Good’ cases than the strict PNL calculation. Importantly, this binary imbalance did not adversely affect the land-mark-localization training of our proposed Attention U-Net. Unlike the baseline classifier, the U-Net is a landmark regression model optimized using Wing Loss to minimize coordinate error rather than binary classification error. Therefore, it learns from the continuous spatial distribution of anatomical features rather than the ratio of ‘Good’ to ‘Poor’ labels, making it robust against the observed class discrepancies. Despite these considerations, the proposed attention regression U-Net model exhibited strong and consistent performance across multiple experimental runs, suggesting its potential for clinical utility.

The between-model differences in landmark-localization error are reported not as clinical endpoints in their own right, but as the mechanistic explanation for the differences in downstream positioning-quality classification, which is the clinically relevant outcome. Because the Good/Poor decision depends on whether the derived posterior nipple line intersects the pectoral muscle line, more accurate localization of the nipple and pectoral endpoints translates into more reliable classification of borderline acquisitions: relative to the plain U-Net, the Attention U-Net improved sensitivity for detecting poorly positioned examinations from 58.6% to 80.2% at comparable specificity, the level at which the improvement carries clinical consequences for repeat imaging and posterior-tissue coverage. The localization errors are an intermediate, technical measure of model precision rather than a clinical positioning-quality metric, and no established clinical threshold defines an acceptable landmark error; their clinical significance is therefore assessed through their effect on the positioning decision rather than in isolation. In routine quality-assurance use, the model functions as decision support: the predicted landmarks, the posterior nipple line, and its intersection are projected onto the image so that the radiographer can verify the result and adjudicate borderline cases, rather than the workflow being governed by a fixed millimetric threshold.

Several studies are noteworthy in the context of DL-based assessment of breast positioning. Brahim et al. [22] employed multiple criteria for MLO-view positioning as outlined by the American College of Radiology and developed a classification DL model to distinguish between poor and good MLO-view positioning. The authors implemented a variety of criteria, such as nipple position, angle of the pectoral muscle, and the PNL, and interconnected individual classifiers for each positioning criterion. Despite achieving an F1 score of 93.3% on a small testing set of 58 mammography examinations, the study’s methodology lacks a detailed explanation regarding the ground-truthing process. Although the authors delineated the criteria used to define positioning quality, they did not provide sufficient details on whether the assessments were qualitative, quantitative, or both. Moreover, while they employed Grad-CAM for explainability, prior research—including our own findings—suggests that saliency methods often lack robustness in mammography tasks [21].

Our study distinguishes itself from Brahim et al. [22] by producing explicit, directly verifiable geometric outputs and by using more robust ground-truthing and evaluation methods. The value of such transparent, verifiable outputs is underscored by findings from Waade et al. [23], who assessed the agreement between commercially available software and radiographers on image positioning. While there was excellent agreement on the PNL in MLO views, discrepancies arose in other criteria, such as the fold in the pectoral muscle and its shape. The authors suggested that the discrepancies might reflect variations in the interpretation or definition of the criteria, or the assessment methods themselves, highlighting the value of transparent, directly verifiable positioning-assessment outputs.

Additionally, Watanabe et al. [24] focused on the nipple profile in their study using a DL method to assess the positioning of MLO views. Their classification model achieved an accuracy of 78%, which is substantially lower than our proposed approach. Like other studies, the transparency of the DL model used in their research was limited.

Ensuring high-quality mammography images is essential for accurate diagnosis, patient outcomes, and workflow efficiency [10]. Designed to act as a “second eye,” this AI-driven model identifies poorly positioned mammograms immediately after acquisition [21]. This real-time feedback not only enables immediate quality control—potentially reducing recall rates—but also serves as an educational tool to help radiographers refine their techniques over time [23]. In screening programs, automated quality assessment can standardize image evaluation, minimize inconclusive exams (e.g., BI-RADS 0), and optimize resource utilization [13,24]. However, challenges such as false positives, potential workflow disruptions, and over-reliance on AI must be carefully managed to ensure seamless clinical integration [13]. While this model aims to support radiographers rather than replace them, further validation and implementation strategies are necessary to maximize its clinical impact.

A methodological consideration is the unit of our cross-validation partitions. In the revised analysis, we used patient-level grouped cross-validation: both MLO views from the same examination were assigned to the same fold, so no patient contributed images to both training and test partitions. This grouped split was stratified on the qualitative Good/Poor positioning label while preserving patient-level grouping. Therefore, the re-vised performance estimates eliminate the potential information leakage that could arise when left and right MLO views from the same examination are separated across folds. Because the evaluation remains internal to a single public dataset, external validation is still required to assess generalizability across populations, scanner vendors, acquisition protocols, and clinical workflows.

Several limitations of the present work must be acknowledged. First, our study is designed as a single-view validation, restricted to evaluating the proposed model’s ability using only MLO views, which, while being the dominant view in mammography due to its comprehensive coverage of breast tissue, does not encompass all diagnostic viewpoints. Future studies are planned to extend our model to include CC views.

Additionally, our assessment primarily utilized the PNL as the criterion for evaluating positioning quality. While the PNL is robust and versatile for MLO views compared to other criteria like the angle and shape of the pectoral muscle, it is important to note that our model might not effectively detect poorly positioned MLO views based on these other criteria. Despite this, our model demonstrated commendable performance, albeit with a slight reduction, when evaluated by an expert breast radiologist. The expert assessed the adequacy of positioning using a broader set of criteria defined by the American College of Radiology, which includes PNL, pectoral muscle shape, and size.

Moreover, all model development and evaluation in this study were internal: performance was estimated by cross-validation within a single dataset, the VinDr-Mammo dataset, collected from two hospitals in Vietnam across three scanner vendors, and no external validation on an independent dataset was performed. The dataset predominantly comprises dense-breast examinations (89.96%), a characteristic more common in Asian populations. Consequently, differences in breast density, anatomy, acquisition protocols, and scanners beyond those represented here may reduce the direct transferability of our findings to other populations and clinical settings [23]. The absence of external validation is a recognized and widespread limitation in radiologic deep learning rather than one specific to this work: only a small minority of diagnostic AI studies report external validation [25], and among those that do, most show at least some degradation of performance on external data [26]. We therefore present these results as a single-dataset, single-view internal validation and regard multi-institutional, multi-vendor, and multi-ethnic external validation, together with prospective in-workflow assessment, as an essential prerequisite before any claim of clinical generalizability.

Additionally, while our dataset split was conducted randomly to capture a representative distribution of cases, we acknowledge that dataset composition and variable distribution could influence model performance. Although a high-level verification (e.g., breast density distribution) indicated no major imbalances, a more granular analysis of clinical outcomes and imaging variations was beyond the scope of this study. Future research should explore whether model performance is affected by variations in data distribution and investigate potential biases across different patient subgroups to further enhance robustness and fairness in clinical applications.

Finally, landmark annotations used to train the regression models were performed by a single board-certified breast radiologist. Although this approach provided internally consistent training labels and follows common practice in mammographic landmark and segmentation studies [27], it precluded estimation of inter-reader and intra-reader variability in defining the nipple centroid and pectoral muscle endpoints. This limitation is mitigated in part by the objective, coordinate-based nature of the ground truth and by the reproducibility of the geometric posterior nipple line, which shows high inter-reader agreement once operationalized [23]; by contrast, qualitative human assessment of the same positioning criterion is considerably more variable [28]. Future studies should employ multiple independent annotators with consensus or adjudication to quantify reader variability and further validate landmark-based positioning assessment.

## 5. Conclusions

In conclusion, this single-dataset, single-view study evaluated an Attention U-Net landmark-regression approach for quantitative assessment of MLO mammography positioning using the PNL criterion. The model provided explicit landmark and PNL outputs and showed improved accuracy and sensitivity compared with a plain U-Net regression baseline and a ResNeXt50 classification baseline. These findings support further external validation and prospective workflow studies to determine whether landmark-based quality-control assistance can improve acquisition quality and reduce repeat imaging.

## Figures and Tables

**Table 1:** Training configuration for the regression and classification models.

Hyperparameter	Regression Models (Attention U-Net, U-Net)	Classification Model (ResNeXt50)
Task	Pec1, Pec2, Nipple landmark localization	Quality classification (Good/Poor)
Optimizer	Adam	Adam
Batch size	8	8
Epochs	300	30
Learning rate schedule	CyclicLR (Base: 1 × 10^−5^, Max: 5 × 10^−4^)	CyclicLR (Base: 1 × 10^−5^, Max: 5 × 10^−4^)
Loss function	Wing Loss (per-landmark weighted)	Cross-Entropy
Pretraining	None (trained from scratch)	ImageNet
Hardware	NVIDIA RTX A6000 GPU	NVIDIA RTX A6000 GPU

**Table 2:** Landmark localization performance: Attention U-Net vs. U-Net (paired comparison, *n* = 2000 MLO-view images pooled across 10 cross-validation folds).

Landmark	U-Net Mean (Std) [mm]	Attention U-Net Mean (Std) [mm]	Mean Diff. (95% CI) [mm]	Wilcoxon *p*	Holm-Adjusted *p*	Cohen’s d
Perpendicular intersection	9.81 (7.06)	5.93 (5.54)	3.88 (3.64, 4.13)	<0.001	<0.005	0.68
Pectoral muscle endpoint 1	8.75 (8.65)	7.15 (7.39)	1.60 (1.33, 1.87)	<0.001	<0.005	0.26
Pectoral muscle endpoint 2	12.86 (12.21)	8.10 (9.93)	4.76 (4.37, 5.15)	<0.001	<0.005	0.54
Nipple	7.02 (5.61)	3.44 (3.89)	3.58 (3.38, 3.78)	<0.001	<0.005	0.80
Angular agreement (°)	3.53 (3.08)	2.89 (2.72)	0.65 (0.54, 0.76)	<0.001	<0.005	0.26

Mean Difference = U-Net error − Attention U-Net error (positive values indicate higher error for U-Net). 95% CI from 10,000-resample bootstrap of paired differences. Wilcoxon: paired signed-rank test, two-sided. Cohen’s d: paired effect size.

**Table 3:** Classification performance of the models against the automated PNL reference (*n* = 2000 MLO-view images pooled across 10 cross-validation folds).

Model	Accuracy (%) [95% CI]	Sensitivity (%) [95% CI]	Specificity (%) [95% CI]
Attention U-Net	85.5 [83.9, 87.0]	80.2 [77.2, 82.9]	88.7 [86.8, 90.3]
U-Net	71.8 [69.7, 73.7]	58.6 [55.1, 62.1]	79.6 [77.2, 81.7]
ResNeXt50	73.7 [71.7, 75.6]	48.2 [44.6, 51.8]	88.9 [87.0, 90.5]

95% confidence intervals computed using the Wilson score interval. Sensitivity is computed for the ‘poor’ class; specificity for the ‘good’ class.

**Table 4:** Classification performance of the models against the expert qualitative reference (*n* = 2000 MLO-view images pooled across 10 cross-validation folds).

Model	Accuracy (%) [95% CI]	Sensitivity (%) [95% CI]	Specificity (%) [95% CI]
Attention U-Net	82.2 [80.5, 83.8]	85.8 [82.6, 88.5]	80.9 [78.8, 82.8]
U-Net	72.4 [70.3, 74.3]	63.1 [59.0, 67.1]	75.7 [73.5, 77.9]
ResNeXt50	78.0 [76.1, 79.8]	55.5 [51.3, 59.6]	86.3 [84.4, 87.9]

Confidence intervals via Wilson score method. Reference labels: holistic clinical assessment by an expert breast radiologist.

**Table 5:** Statistical comparison between models (fold-level paired analysis). (**a**) Automated PNL reference. (**b**) Expert qualitative reference.

(a)								
Comparison	Metric	Model A Mean ± Std [95% t-CI]	Model B Mean ± Std [95% t-CI]	Wilcoxon *p*	Holm-Adjusted *p*	Cohen’s d	McNemar *p*	Holm-Adjusted *p*
Attention U-Net vs. U-Net	Accuracy	0.86 ± 0.02 [0.84, 0.87]	0.72 ± 0.04 [0.69, 0.75]	0.002	0.008	2.58	<0.001	<0.001
	Sensitivity	0.80 ± 0.04 [0.77, 0.83]	0.59 ± 0.09 [0.52, 0.65]	0.002	0.016	1.86	—	—
	Specificity	0.89 ± 0.03 [0.87, 0.91]	0.80 ± 0.05 [0.76, 0.83]	0.006	0.023	1.32	—	—
Attention U-Net vs. ResNeXt50	Accuracy	0.86 ± 0.02 [0.84, 0.87]	0.74 ± 0.05 [0.70, 0.77]	0.002	0.008	2.26	<0.001	<0.001
	Sensitivity	0.80 ± 0.04 [0.77, 0.83]	0.48 ± 0.10 [0.41, 0.55]	0.002	0.016	2.57	—	—
	Specificity	0.89 ± 0.03 [0.87, 0.91]	0.89 ± 0.04 [0.86, 0.92]	0.767	0.767	−0.07	—	—
U-Net vs. ResNeXt50	Accuracy	0.72 ± 0.04 [0.69, 0.75]	0.74 ± 0.05 [0.70, 0.77]	0.106	0.211	−0.25	0.21	0.21
	Sensitivity	0.59 ± 0.09 [0.52, 0.65]	0.48 ± 0.10 [0.41, 0.55]	0.037	0.111	0.75	—	—
	Specificity	0.80 ± 0.05 [0.76, 0.83]	0.89 ± 0.04 [0.86, 0.92]	0.010	0.039	−1.30	—	—
(**b**)								
**Comparison**	**Metric**	**Model A Mean ± Std [95% t-CI]**	**Model B Mean ± Std [95% t-CI]**	**Wilcoxon** ***p***	**Holm-Adjusted** ***p***	**Cohen’s d**	**McNemar** ***p***	**Holm-Adjusted** ***p***
Attention U-Net vs. U-Net	Accuracy	0.82 ± 0.03 [0.80, 0.84]	0.72 ± 0.03 [0.70, 0.75]	0.004	0.008	1.65	<0.001	<0.001
	Sensitivity	0.86 ± 0.07 [0.81, 0.91]	0.63 ± 0.11 [0.55, 0.71]	0.002	0.016	2.05	—	—
	Specificity	0.81 ± 0.03 [0.79, 0.83]	0.76 ± 0.03 [0.74, 0.78]	0.110	0.219	0.64	—	—
Attention U-Net vs. ResNeXt50	Accuracy	0.82 ± 0.03 [0.80, 0.84]	0.78 ± 0.03 [0.76, 0.80]	0.010	0.010	1.13	<0.001	<0.001
	Sensitivity	0.86 ± 0.07 [0.81, 0.91]	0.56 ± 0.09 [0.49, 0.62]	0.002	0.016	2.21	—	—
	Specificity	0.81 ± 0.03 [0.79, 0.83]	0.86 ± 0.04 [0.84, 0.89]	0.006	0.023	−1.14	—	—
U-Net vs. ResNeXt50	Accuracy	0.72 ± 0.03 [0.70, 0.75]	0.78 ± 0.03 [0.76, 0.80]	0.049	0.146	−0.88	0.008	0.008
	Sensitivity	0.63 ± 0.11 [0.55, 0.71]	0.56 ± 0.09 [0.49, 0.62]	0.192	0.219	0.49	—	—
	Specificity	0.76 ± 0.03 [0.74, 0.78]	0.86 ± 0.04 [0.84, 0.89]	0.002	0.018	−1.65	—	—

(**a**) Cochran’s Q (3 models, automated reference): Q = 166.5, *p* < 0.001. (**b**) Cochran’s Q (3 models, expert qualitative reference): Q = 73.5, *p* < 0.001. 95% t-CI: t-distribution confidence interval over 10-fold means (df = 9). Wilcoxon: paired signed-rank test on fold-level metrics; minimum two-sided *p* at *n* = 10 is 2/2^10^ ≈ 0.00195. Cohen’s d: paired effect size on fold differences (Model A − Model B); positive values favor Model A. McNemar *p*: per-image test on classification correctness (*n* = 2000); reported only for accuracy as the test aggregates over the full test set and is not separately defined for class-specific metrics; dash (—) indicates not applicable.

## Data Availability

The data labels and code related to this study are made publicly available to support the research community. Researchers can access these resources via the following GitHub repository: https://github.com/enescanerkan/deep-breast-positioning, accessed on 1 June 2026. The repository provides data annotations and code that can be utilized and adapted for specific research purposes, enabling the exploration of new methodologies and advancements in related fields.

## Supplementary Materials

The following supporting information can be downloaded at: https://www.mdpi.com/article/10.3390/diagnostics16142262/s1, Figure S1: Comparison of our approach using attention U-Net with the Grad-CAM results of the baseline classification model on a single patient’s two MLO views (1). True-positive and true-negative examples of attention U-Net (2). Examples of our attention U-Net and the Grad-CAM results of the baseline classification model (3); Figure S2: True-positive and true-negative examples of attention U-Net; Figure S3: Examples of our attention U-Net and the Grad-CAM results of the baseline classification model.

## Abbreviations

The following abbreviations are used in this manuscript:

CC	Craniocaudal
MLO	Mediolateral oblique
DL	Deep learning
PNL	Posterior nipple line
XAI	Explainable AI
Grad-CAM	Gradient-weighted class activation mapping
