# Supplementary material for: Landmark Regression with Attention U-Net for Assessing Breast Positioning Quality in MLO Mammography View
Source: Diagnostics (Basel). 2026 Jul 20;16(14):2262. doi: 10.3390/diagnostics16142262 (PMC13409498; doi:10.3390/diagnostics16142262)
Supplement: Supplementary file 1 [file diagnostics-16-02262-s001.zip › diagnostics-4389017-supplementary.pdf]

Supplementary Files

Figure S1: Comparison of our approach using attention U-Net with the Grad-CAM results of the baseline classification model on a single patient's two MLO views.

Figure S2: True-positive and true-negative examples of attention U-Net.

Figure S3: Examples of our attention U-Net and the Grad-CAM results of the baseline classification model.

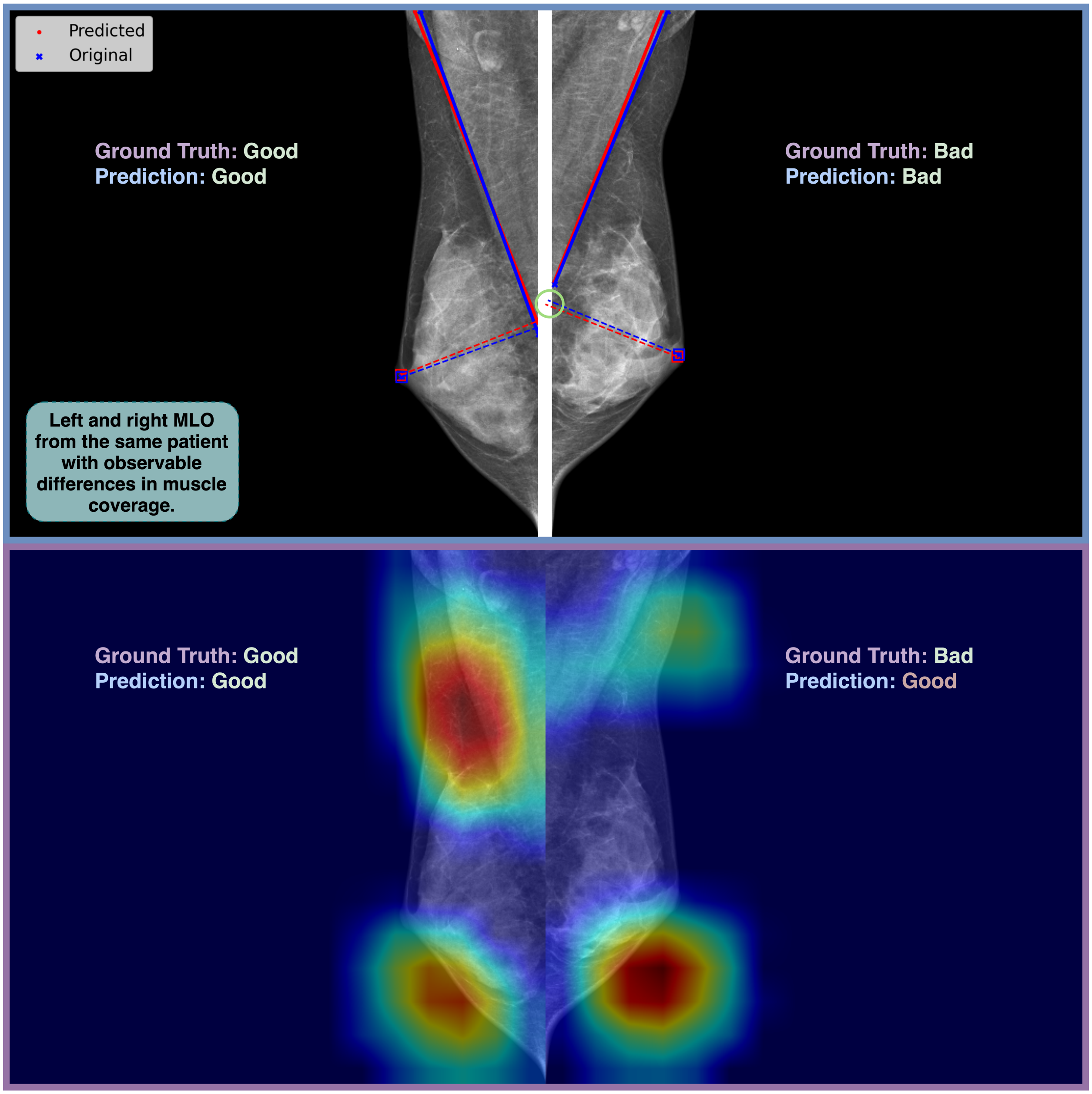

Figure S1. Comparison of our approach using attention U-Net with the Grad-CAM results of the baseline classification model on a single patient's two MLO views.

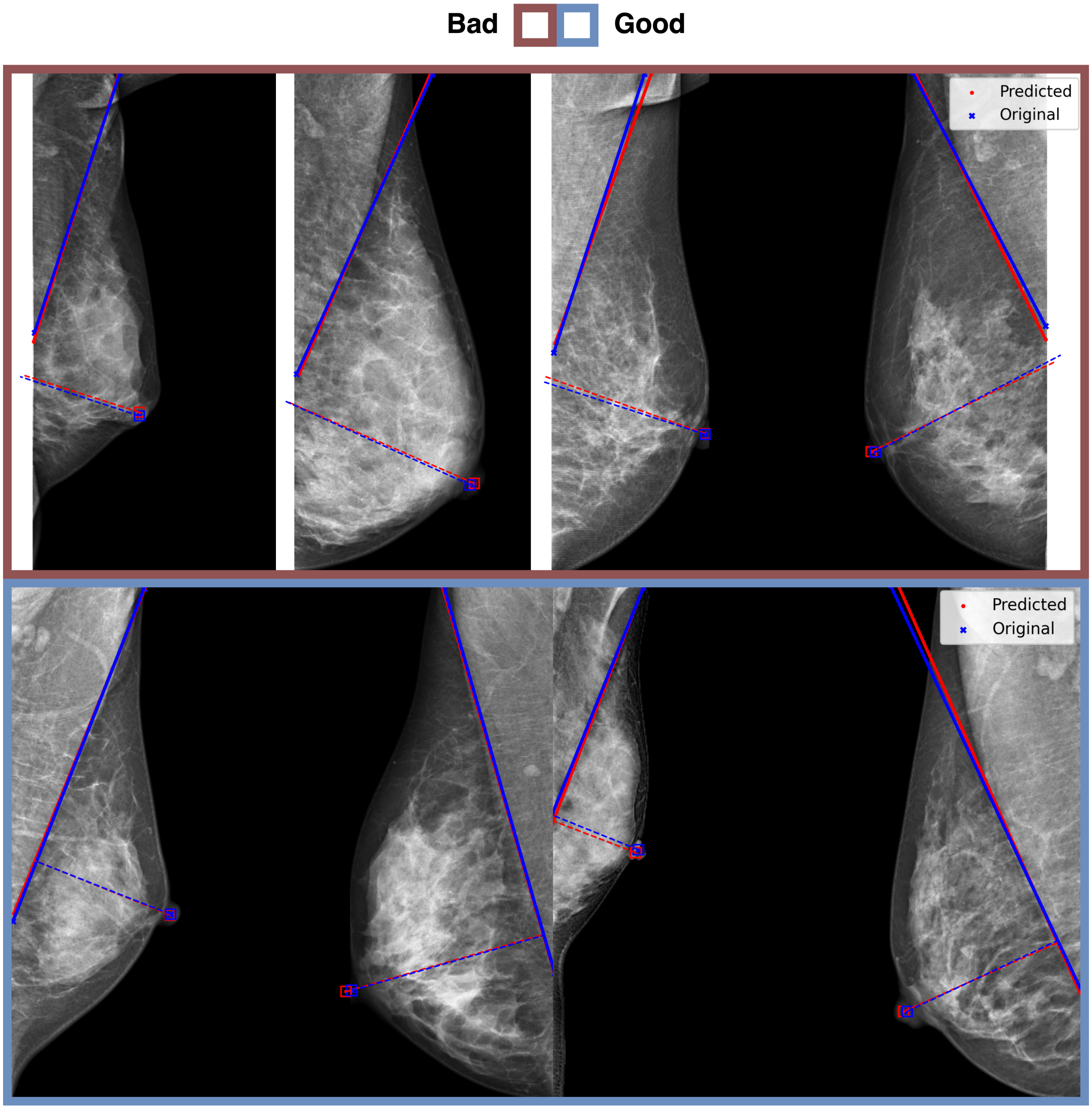

Figure S2. True-positive and true-negative examples of attention U-Net.

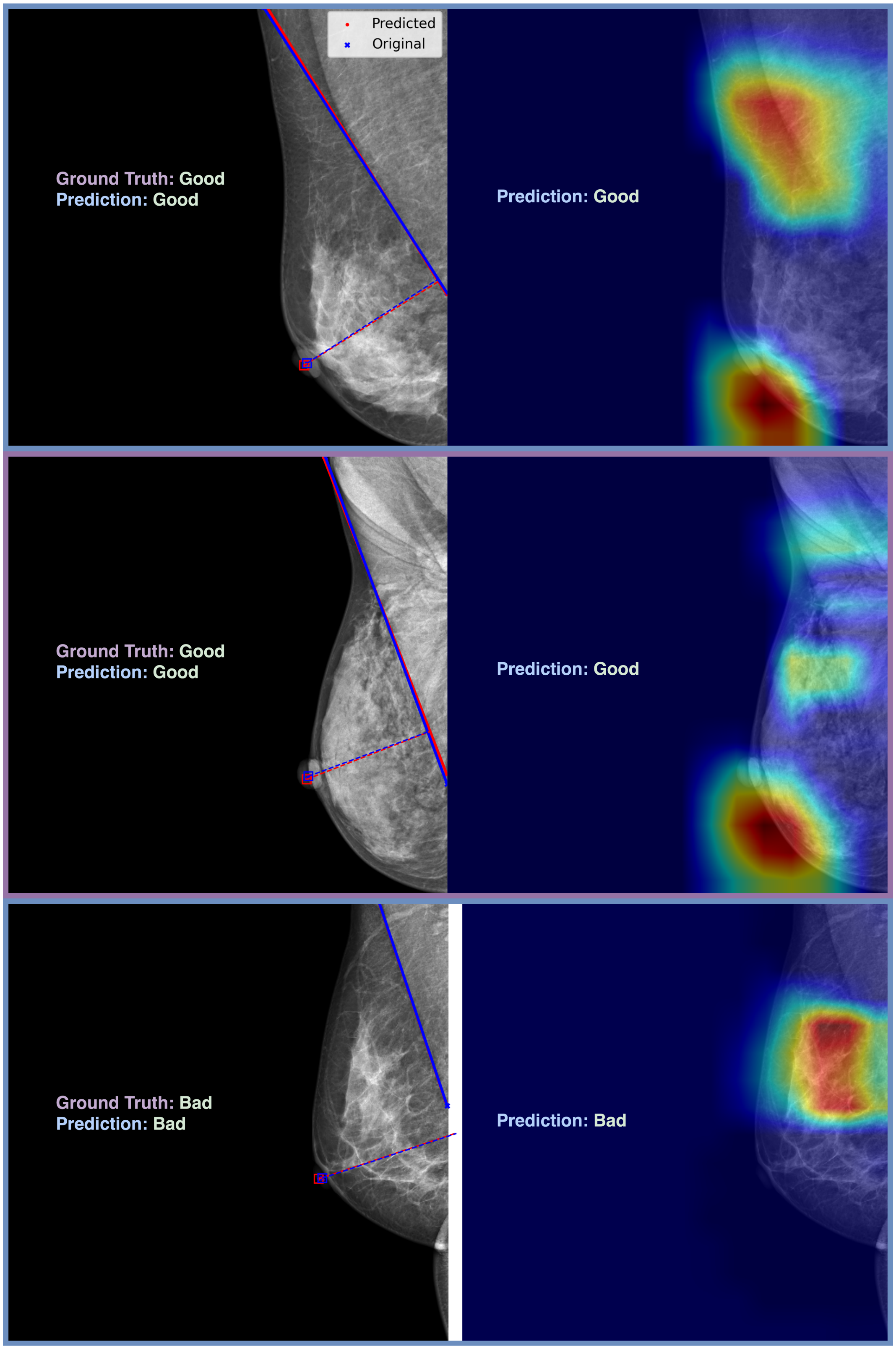

Figure S3. Examples of our attention U-Net and the Grad-CAM results of the baseline classification model.
